# Three-dimensional chemotaxis-driven aggregation of tumor cells

**DOI:** 10.1038/srep15205

**Published:** 2015-10-16

**Authors:** Alberto Puliafito, Alessandro De Simone, Giorgio Seano, Paolo Armando Gagliardi, Laura Di Blasio, Federica Chianale, Andrea Gamba, Luca Primo, Antonio Celani

**Affiliations:** 1 Candiolo Cancer Institute FPO-IRCCS, Candiolo, Turin, Italy; 2 Swiss Institute for Experimental Cancer Research (ISREC), School of Life Sciences, Swiss Federal Institute of Technology (EPFL), Lausanne, Switzerland; 3 Edwin L. Steele Laboratory for Tumor Biology, Harvard Medical School, Massachusetts General Hospital, Boston, MA, USA; 4 Department of Oncology, University of Turin, Turin 10060, Italy; 5 Institute of Condensed Matter Physics and Complex Systems, Department of Applied Science and Technology, Polytechnic University of Turin, Corso Duca degli Abruzzi, 24, 10129 Torino, Italy; 6 Human Genetics Foundation (HuGeF), Via Nizza 52, Torino, Italy; 7 Istituto Nazionale di Fisica Nucleare (INFN), Torino, Via Giuria 1, 10125 Torino, Italy; 8 Quantitative Life Sciences Unit, The Abdus Salam Center for Theoretical Physics (ICTP), Strada Costiera 11, I-34151 Trieste, Italy

## Abstract

One of the most important steps in tumor progression involves the transformation from a differentiated epithelial phenotype to an aggressive, highly motile phenotype, where tumor cells invade neighboring tissues. Invasion can occur either by isolated mesenchymal cells or by aggregates that migrate collectively and do not lose completely the epithelial phenotype. Here, we show that, in a three-dimensional cancer cell culture, collective migration of cells eventually leads to aggregation in large clusters. We present quantitative measurements of cluster velocity, coalescence rates, and proliferation rates. These results cannot be explained in terms of random aggregation. Instead, a model of chemotaxis-driven aggregation – mediated by a diffusible attractant – is able to capture several quantitative aspects of our results. Experimental assays of chemotaxis towards culture conditioned media confirm this hypothesis. Theoretical and numerical results further suggest an important role for chemotactic-driven aggregation in spreading and survival of tumor cells.

Multiple acquired genetic mutations drive the gradual alteration of normal growth control mechanisms that leads to cancer. Growth regulation in healthy individuals is realized through the controlled cellular response to different stimuli such as growth factors, cell-matrix or cell-cell contact. These components can be turned into mediators of unrestrained cell proliferation through either autocrine or paracrine mechanisms^1^,^2^,^3^.

Once a tumor starts growing, other cellular functions become decisive for the tumor to outcompete the neighboring normal cells and ultimately evade the primary site. One of such functions is the cell’s ability to move in response to stimuli: indeed the migratory machinery is often found to be altered in tumors^4^,^5^,^6^,^7^, and can be exploited by tumor cells to increase survival probability or gain selective advantage^8^,^9^,^10^. Furthermore evidence pointing at tumor invasion and metastasis as an analogous of normal morphogenesis is compelling^11^,^12^.

About 90% of human cancers are carcinomas, i.e. malignancies originating from epithelial tissues, and a widely accepted view of tumor progression in carcinomas involves the growth of a tumor *in situ* followed by a transformation of cells which undergo an epithelial to mesenchymal transition (EMT)^3^. Isolated highly motile tumor cells are then able to move and spread throughout the entire body depending on the matching between their transcriptional background and/or acquired genetic alterations and the visited environment.

However cancer cells can migrate as collective units^13^,^14^,^15^,^16^. While *in vivo* evidence for isolated migrating cancer cells has been elusive, several indisputable examples have been provided to show that cells move as groups both in normal development and in cancer models^17^,^18^,^19^,^20^. The molecular fingerprints of these phenotypes are not well defined yet and are thought to be partially overlapping with innate abilities of epithelial cells^14^,^21^,^22^, indicating that cancer invasion by collective migration might not require the complete loss of epithelial markers.

An important question regarding collective migration is whether it can confer a selective advantage as opposed to pure mesenchymal migration. In principle, aggregation of cells into clusters might represent a selective advantage over single cells in many different ways^23^. The capability of tumor cells of moving as a cluster has been related to the ability to escape certain facets of the immune response and to be advantageous after extravasation, where adhesion-dependent signaling is not present and mechanical strain can be relevant^8^,^24^,^25^. For instance, homotypic aggregation of tumor cells has been described previously to be of paramount importance in the development of breast cancer as it might prevent anoikis^26^. Recently, it has been demonstrated that multicellular aggregates can form from heterogeneous cancer cell populations at the primary site. These clusters can be detected in the bloodstream and, albeit more rare than single cells, have much higher morbidity^27^. Here, we report a novel growing phenotype found in a cancer cell (CC) line. Cells seeded in three dimensional BME gels as single cells are able to grow as cluster and move towards each other. Close clusters aggregate into larger clusters and cluster velocity and proliferation depend on cell density. We present quantitative measurements of aggregation dynamics, rates of proliferation and velocity of clusters. Our experimental results indicate that the cell seeding density influences the average velocity of cell migration, but not the overall time-scale of the aggregation process. This observation is in striking contrast with what would be expected if aggregation was due to random, undirected motion. In this latter case one would observe density-independent migration rates – as clusters would move independently of each other – and a speed-up of aggregation with increasing number density – as the cluster-cluster encounter rate would be higher. Our results seem instead to point at some “action at a distance” between clusters at the origin of the coalescence process.

What drives aggregation then? How is it possible that aggregation rates are independent of density? To answer these questions we formulated the theoretical hypothesis that cells or cell clusters attract each other by following a gradient of a diffusible factor. The model of chemotaxis driven cell aggregation (CDA) explains our quantitative measurements and allows to predict several distinctive dynamical features. We discuss the experimental results and predictions in view of the potential advantage that homotypic aggregation might have in tumor spreading and survival. Theoretical hypotheses and approximations are then confirmed by means of numerical simulations and corroborated by further experimental evidence.

## Materials and Methods

### 3D cell culture

Cell lines were cultured in RPMI media (PC3, DU145, LnCaP) or DMEM (MDA-MB-231) supplemented with 10% Fetal Bovine Serum, Penicillin/Streptomycin and L-Glutamine. Cells were kept at 37 °C under 5% CO2 humidified air. For 3D cell culture cells were either trypsinized, diluted to the desired concentration and directly seeded in BME gel or preaggregated overnight in low cell attachment plates with 1% methylcellulose solution. BME gel was prepared as follows: a layer of Growth Factor Reduced (GFR) Matrigel (100 *μ*L, rediluted to a final concentration of 8 mg/mL) was deposed on the bottom of a standard 48-well plate. A second layer of GFR-Matrigel (same amount and concentration), pre-mixed with single cells or aggregates, was deposed on the top of the previous layer, and a third layer of GFR-Matrigel was deposed on top of these two (same amount and concentration). All operations previous to polymerization were conducted on ice to avoid BME solidification. After polymerization, each well was filled with 500 *μ*L culture media (M199 with 10% FBS,Pen/Strep and L-Glutamine). The plate was then kept in the incubator and media were replaced daily. Timelapse videomicroscopy experiments were conducted on a standard inverted bright-field microscope equipped with a motorized stage and an incubator to keep the plate stably at 37° and 5% CO2. For longer timelapse experiments, cells were kept in the incubator and imaged once a day for 20 or more days.

### Proliferation assay

**Figure 1:**
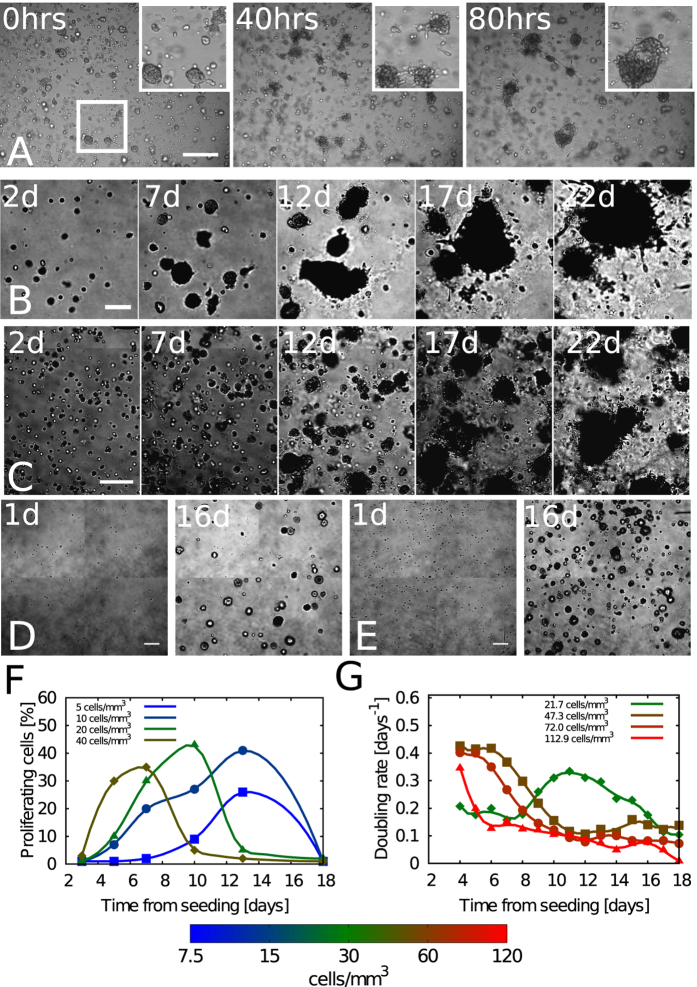
CC spheroids grow, migrate and aggregate in BME embedded 3D cultures. (**A**) PC3 cell spheroids were obtained under overnight culture in low-attachment conditions. Small heterogeneous clusters were then embedded in BME gels. Cells were then imaged by means of time-lapse imaging and representative snapshots at 0, 40 and 80 hours are shown in the first row of images. The images show clusters that emit protrusions and move toward each other forming larger clusters. The white square in the middle of the leftmost panel is enlarged in the inset at the top right corner of the three snapshot to illustrate one aggregation event. Scale bar is 200 *μm*. (**B**,**C**) The second and third row show snapshots of time-lapse images at indicated times obtained by single-cell seeding of PC3 embedded into BME at low and high density respectively. Seeding densities calculated *a posteriori* are 35 and 113 cells/*mm*^3^ respectively. Single cells move and grow as clusters that merge into larger clusters. Scale bar is 200 *μm*. (**D**,**E**) DU145 cells were seeded at 23 and 77 cells/*mm*^3^. While cells do grow as spheroids, they do not move or aggregate. Rare merging events are observed whenever clusters come into contact by pure growth. (**F**) The fraction of proliferating cells was measured by means of an EdU-CLICK assay. Each curve represents, for each given seeding density, the percentage of EdU positive cells at each time of the experiment. Densities are 5 (blue squares), 10 (dark blue circles), 20 (green triangles), 40 (brown diamonds) cells per *mm*^3^. A peak of proliferating cells occurs at different times depending on seeding density. In wells where the seeding density was high the proliferation peak is reached before than wells where cells are sparse. The color-bar is common to panels (**F**,**G**). (**G**) To estimate cells doubling times we performed growth measurements on clusters by means of image analysis. Measured densities are 21.7 (green diamonds), 47.3 (brown squares), 72.0 (dark red circles), 112.9 (red triangles) cells per *mm*^3^. Indeed cluster size doubling time has a peak occurring at different time from seeding depending on seeding density and peak times are similar to those found in panel F. Denser clusters tend to reach the peak faster than sparse clusters.

Data from Fig. 1F were generated by applying Click-It Edu technology (Lifetechnology) to 3D cell culture. Briefly, BME-embedded cells were fed with the intercalating agent (EdU) for 6 hours at indicated seeding time. BME was then dissolved by incubating with Cell Recovery Solution (BD) and cells were then fixed in 4% PFA, stained with the EdU detection reagent and analyzed by flow cytometry. The measured fraction of cells positive for EdU incorporation is reported, corresponding to the proliferating population.

### Chemotaxis assay

Chemotaxis assays were performed with Transwell Permeable Supports (BD) culture plate inserts with 8 *μ*m pore size.

30000 cells were plated on top of the insert in 400 *μL* of media (serum free or other as indicated). The lower chamber was filled with 750 *μL* of conditioned media (or other, as indicated). After 24 hours, cells sitting on top of the insert were scraped and cells sitting on the bottom of the insert were then fixed with 2.5% Glutaraldehyde and stained with crystal violet in methanol. Images were acquired on an upright microscope and analyzed by means of a custom written Matlab algorithm. Each experiment was conducted 3 times with at least 2 technical replicates and different batches of conditioned media were used.

### Image processing and quantifications

Digital images were processed by custom computer algorithms written in Matlab to extract quantitative data on cell aggregation, proliferation and velocity^28^. To extract cluster size, images were first corrected for non uniform illumination. To this end, unprocessed images were convoluted with a flat kernel to obtain a background image. Then the original image was divided by the background to obtain a corrected image. Afterwards, to equilibrate differences in the intensity due to out-of focus effects, intensity profiles were centered around the mean and used as the argument of a hyperbolic tangent. At this point, single cells were detected by high-pass filtering images while large clusters were simply identified by thresholding was used to obtain a binary image, which was then labeled and from which quantitative data could then be extracted.

To obtain cell or cluster velocities we made use of a tracking technique frequently used in hydrodynamics called Particle Image Velocimetry or PIV. Briefly, cells or clusters were identified either automatically or semi-automatically. A small image tile containing the object to track is cropped from the original image and the best match in the next frame is found by computing spatial cross-correlation function.

### Data analysis

Data from Fig. 1G were obtained by calculating the total area of clusters in a field of view over time, computing the time derivative of the logarithm and multiplying by 1.5 to account for the fact that growth is proportional to the volume of clusters and not surface.

**Figure 2:**
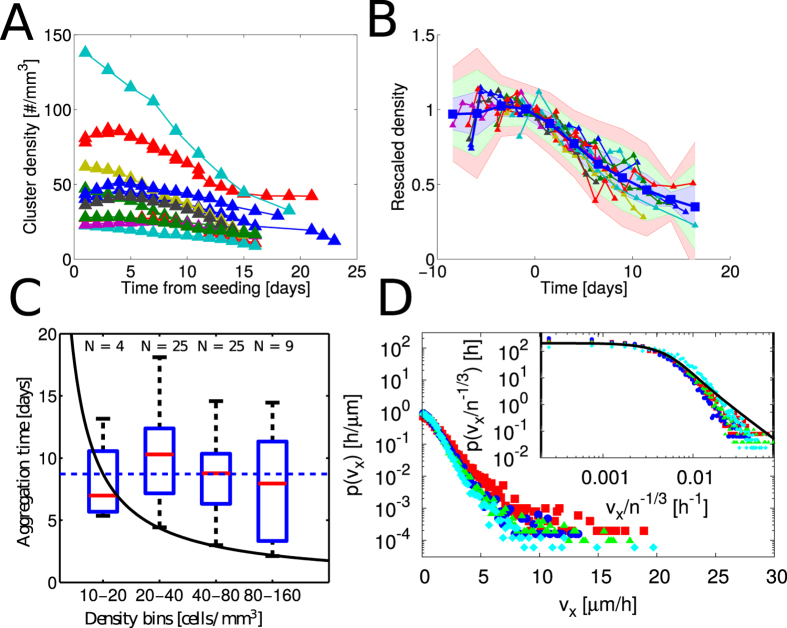
Dynamics of CC spheroids aggregation. (**A**) The number of objects in the image (either cells or clusters) was measured to calculate density at any time of time-lapse movies of the aggregation assays. Semi-automatic image analysis methods were used to count the number of objects. Each curve represents a different independent experiment. (**B**) Aggregation curves were fitted independently, rescaled and time-shifted with the values of the obtained parameters. Curves are time-shifted depending on the value of the fit of the lag-times, and rescaled according to the fitted seeding density. Shades indicate 1*σ* (blue), 2*σ* (red), 3*σ* (green). (**C**) Aggregation times, i.e. halving time of the number of objects in each field of view was measured for several densities (N = 63). The blue dotted line is the median of the data, *τ*_2_ = 8.93 days, plotted as a guide to the eye. Each box is the box-plot of the data contained in the corresponding logarithmic bin of cell density. Red lines are medians, blue boxes are 25th and 75th percentiles, and whiskers are the most extreme points. The continuous black line represents the function *x*^−1^, plotted here as a reference, as this would be the dependence of the time from density in the case of pure random aggregation. (**D**) Velocity of cells/clusters measured at early time-points of the aggregation assay. Densities are 21 cells/*mm*^3^ (red squares), 31 cells/*mm*^3^ (blue circles), 59 cells/*mm*^3^ (green triangles) and 189 cells/*mm*^3^ (cyan diamonds). Here *p*(*v*_*x*_) indicates the probability density function (PDF) of the variable *v*_*x*_. The PDF of the components *v*_*x*_ and *v*_*y*_ of the velocity was found to have heavy tails (power law decay) and to be function of the seeding density. Denser cells move slower on average than sparse cells. The experimental velocity distribution is compared with the predicted Holtsmark distribution (solid line) with width v_*_ = 2.8 · 10^−3^ *n*^−1/3^ where *n* is the cluster density.

To fit and rescale the data on cell aggregation shown in Fig. 2, curves for each aggregation assay were fitted to: 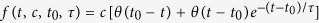, where *θ* is the Heaviside function. Once fitted, curves were rescaled to obtain Fig. 2B and halving times of clusters calculated as 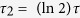 and shown in Fig. 2C.

**Figure 3:**
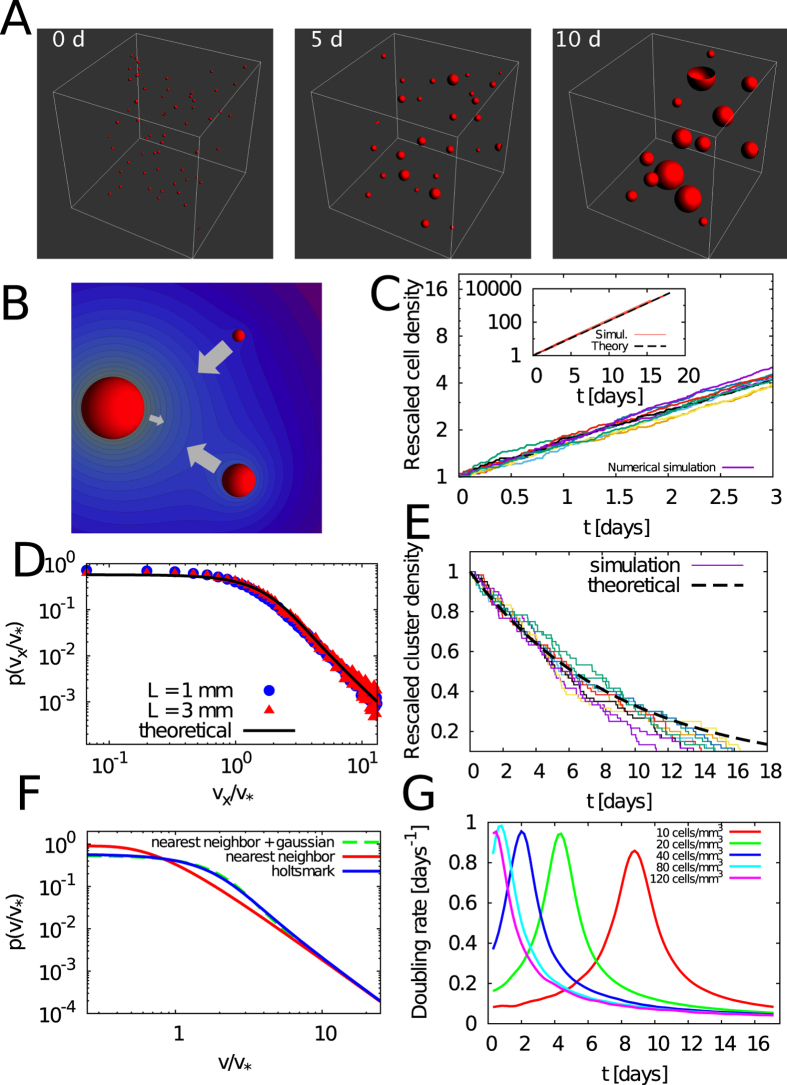
Theoretical and Computational results on chemotaxis driven cell aggregation. (**A**) Computational model of chemotactic driven aggregation. The simulation volume is seeded with clusters (in red) that proliferate with a constant growth rate and attract each other by a diffusible chemoattractant as described in eq. (1). The clusters are spherical and coalesce upon boundary contact. Periodic boundary condition are considered for cluster position and chemoattractant concentration field. The simulation parameters are summarized in Table 1. (**B**) Cartoon illustrating three clusters of different sizes attracting each other along the direction of the arrows. The thickness of the arrows indicate the magnitude of the velocity of each clusters while the blue color-map indicate the concentration field of the chemoattractant in the space (increasing concentration from dark to light). (**C**) Rescaled density of cells in all clusters as function of time. Several different simulations are shown to illustrate the effect of different initial seeding configurations (while keeping the density constant) on the total growth. (inset) The prediction of the theoretical model (black dashed line) is compared with the results of the computational model (in red). Results from independent simulation runs with the same set of parameters are shown. (**D**) Simulated cluster velocity PDF. The cluster velocity PDF at the simulation start (blue circles) agrees with the predicted Holtsmark distribution (black solid line). A slight discrepancy for intermediate values is due to finite volume effects and is reduced for a larger simulation volume (red triangles). (**E**) Rescaled density of clusters as function of time. The prediction of the theoretical model (dashed black line) is compared with the results of the computational model (continuous colored lines). Results from independent simulation runs with the same set of parameters are shown. (**F**) The Holtsmark distribution arises from the sum of two approximately independent contributions. The power-law behavior for large velocities is produced by the interaction with the nearest cluster (in red). The interaction with the farther clusters contributes with a Gaussian term for low and intermediate velocities. The sum of the two contributions (dashed green) agrees with good approximation with the exact Holtsmark distribution (in blue). (**G**) To explain the patterns of cell proliferation observed in Fig. 1G, we hypothesized that proliferation depends on the concentration of a secreted factor (see main text or SI). The values of density used in the simulations are 10 (red), 20 (green), 40 (blue), 80 (cyan), 120 (magenta) cells/*mm*^3^. The plot presents the results of several numerical simulations for different initial densities, showing a peak of proliferation that depends on initial density, analogously to Fig. 1G.

The theoretical cell/cluster velocity distribution (Fig. 2D) is obtained by a simultaneous fit of the velocity distributions for all cluster densities (quantile method). To generate data from Fig. 3G we ran a simulation with the same parameters of Table 1 and hypothesized that each cell in a cluster proliferates with a rate: 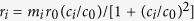, where *m*_*i*_ is the number of cells in the cluster *i, c*_*i*_ is the total concentration felt by the cluster, *c*_0_ is a reference concentration. Below *c*_0_ the proliferation response decreases with the ligand concentration, while above *c*_0_ it decreases (*c*_0_ = 10^9^ *mm*^−3^) and *r*_0_ is the basal cell proliferation rate, chosen to be 3 days^−1^. The dependence of the proliferaton rate from the concentration of the chemoattractant is justified by the data presented in Fig. 1F,G, where higher seeding density (i.e. higher concentrations) are correlated with higher proliferation. The doubling rate is defined (analogously to Fig. 1G) as 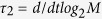.

## Results

### BME embedded CCs grow into larger aggregates in culture

We first assessed the ability of a human prostate carcinoma cell line with high metastatic potential, the PC3 cell line, to grow and invade when embedded in BME gels. Spheroids of PC3 were generated by growing cells under non-adherent culture conditions and then embedded, resulting in multicellular aggregates in BME gels. This procedure generates a spatially uniform distribution of clusters of heterogeneous size (See SI Movie 1). Both single cells and cell clusters can move within the matrix by developing protrusions. Typical observed velocities are of the order of 1 *μ*m/h. By time-lapse microscopy we recorded the behavior of cells at longer times and observed that clusters tend to aggregate into larger structures on timescales of the order of several days. Aggregation occurs through long protrusions and is followed by a reshaping of the aggregated cluster into a new cluster of round shape. Snapshots of aggregation events are shown in Fig. 1A–C.

To gain further insights into the dynamics of this process we performed several time-lapse experiments by seeding cells as single-cell suspensions at different initial densities (see SI Movie 2). Single cells start growing after a short lag-phase (1–2 days), with duplication times of the order of 2 days. Some clusters actively move either in an apparently random direction or toward other clusters. After several days clusters that do not touch each other produce protrusions that allow aggregation. Aggregation of larger clusters (up to several hundreds of *μ*m) is also observed. At the end of the culture cell aggregation slows down and eventually stops.

Notably, the ability to aggregate in 3d culture is not common to all CC lines, as shown in Fig. 1D and SI Movie 3–4. For example DU145 and LnCaP cells, which both grow into clusters when embedded in BME gels, are not able to aggregate. Interestingly, these two CC lines are less metastatic compared to PC3 cells^29^. The same phenomenon was observed in MDA-MB-231 cells, a well characterized breast cancer cell line with invasive and metastatic ability (see Fig. SI2C and SI Movie 5).

### Cell proliferation and cluster growth rates

Cells proliferate when embedded, as can be clearly seen by looking at the size of clusters shown in Fig. 1B. To analyze the relationship of cell proliferation on seeding density and time we performed a 5-ethynyl-2′-deoxyuridine (EdU) incorporation assay on aggregating cells (see Fig. 1F). The number of proliferating cells reaches a peak after several days in culture. The peak occurs earlier for higher seeding density and later when cells are sparsely seeded. We also measured the cluster sizes through quantitative image analysis to investigate whether the clusters’ volumes followed a similar time-course. As shown in Fig. 1G, we indeed found that large seeding densities induce high volumetric growth-rates earlier than low densities.

### Aggregation rates are independent of seeding density

To better characterize aggregation and its cause, we measured the aggregation rates for several runs at different seeding densities. Results are shown in Fig. 2A–C. Aggregation rates are essentially independent of initial density, i.e. the number of clusters halves in about 9 days for all densities we considered. Initial densities range from 10 to 200 cells per mm^3^. This choice is motivated by the fact that too sparse cells do not aggregate, presumably because they do not grow well and are too far away from each other (on average 500 *μm*). On the other hand, when cells come too close initially (below 100 *μm*), they tend to spontaneously aggregate as soon as they touch each other and it is difficult to separate active aggregation from contact. We could not measure aggregation beyond three weeks of time as at this stage cells proved to suffer culture conditions and we could not detect well all the structures present in the images. Indeed single cells started to detach from clusters and basically all the field of view was covered with clusters or cells.

### Velocity distributions depend on number cell density

Cell velocity was measured in early stages of aggregation, after seeding. PIV techniques were used to measure the velocity distribution of cells for different seeding densities (see Materials and Methods and SI text for further details). As shown in Fig. 2D, the distribution of velocities has a large core indicating that a large fraction of cells move at slow speeds. However, a consistent number of events also occurs at values far beyond the standard deviation. We will show below how this is related to the kinetics of aggregation. As displayed in Fig. 2D lower densities are associated to larger velocities.

### Theoretical model of aggregation

We noticed that the experimental results could not be explained by coalescence under random, independent motion of clusters. Indeed, the dependence of the velocity on the initial seeding density rules out the possibility that speeds of different clusters be independent and suggests the existence of an interaction between them. Moreover, the observation that aggregation rates are independent of seeding density is in stark contrast with the results expected for random aggregation. In that case, rates are proportional to the initial number density: the more closely packed the clusters are, the faster the coalescence process. Therefore, we explored the possibility that the interaction between clusters be mediated by a secreted, diffusible attractant as sketched in Fig. 3A,B. Since the time needed for molecules to diffuse across the domain is much faster than cell movement, the secreted factor builds up a quasi-steady concentration profile peaked around each cluster. Concentration then decays as the inverse of the distance from the center up to an interaction length-scale 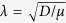, set by the diffusion coefficient *D* of the diffusible factor and its degradation rate, *μ*, where it starts falling off exponentially fast. Reasonable values for these two parameters are *D* = 10 – 20 *μ*m^2^/s for *D* and *μ*^−1^ = 0.5 ÷ 2 days, yielding an interaction length-scale of approximately 1 mm. We therefore predict that chemotaxis-driven aggregation should cease whenever initial density is smaller than a few cells per cube millimeter. Indeed on the timescale of the experiments we performed (between 10 and 20 days), we could not observe aggregation taking place below a seeding density of around 10 cells/*mm*^3^.

Within the interaction length-scale, because of the slow power-law decay of the concentration profile (inversely proportional to the cluster-cluster distance), the mean concentration of chemoattractant receives contributions by all clusters. As a result, the average level of diffusible factor generated by clusters is spatially uniform and proportional to the production rate of chemoattractant, to the total number of cells and inversely proportional to the degradation rate of the chemoattractant (detailed calculations are presented in SI).

The molecular mechanism by which cells feel the concentration field and orient their motion is the spatially asymmetric activation of cell surface bound receptors by the diffusible ligand. The strength of directional signaling (resulting in the orientation and magnitude of migration velocity) can be assumed to be proportional to the magnitude of the gradient of the bound receptors density, which is in turn proportional to the concentration gradient^30^,^31^. For a general monovalent ligand-receptor system, taking into account endocytosis and recycling, the surface density of ligand-receptor complexes can be calculated and yields:
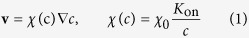
where **v** is the velocity of cells or clusters, *c* is the concentration of chemoattractant, ∇*c* its gradient, *χ*(*c*) is the chemotactic coefficient, *χ*_0_ is a reference chemotactic responsivity and *K*_on_ is a reference concentration. This expression holds in a range of concentrations between two limits *K*_on_ and *K*_off_ that are functions of the kinetic parameters of the model (see SI). In this range cells respond to fold-changes in concentration, a behavior known as Weber-Fechner law^32^.

A further consequence of the slow decay of the concentration profile generated by a single cluster is that gradients are largely dominated by the contribution of the nearest cluster and interactions between clusters are essentially pairwise (detailed calculations are included in the SI text). Thus large values of the concentration gradient come from the action of nearest-neighbors while low and intermediate values come from the contribution of farther clusters. From eq. (1), and the fact that the background concentration is spatially uniform, it immediately follows that the velocity at any given point is statistically distributed in the same way as the concentration gradient. Thus fast migration events are determined by close clusters that aggregate while lower and intermediate velocities are on average originating from the distribution coming from all clusters. A detailed calculation shows that the statistics of cluster velocity follows the Holtsmark distribution^33^, which has heavy power tails *p*(*v*) ~ *v*^−5/2^ and width v_*_ ~ *n*^−1/3^ where *n* is the density of clusters and *v* one of the components of the velocity (see SI). This distribution also arises in the study of the gravitational field generated by a random distribution of masses as well as for the electric field generated by many randomly-distributed charged particles (see^34^ and refs therein). Remarkably, our general model thus predicts the same dependence velocity/density obtained in the experiments, as shown in Fig. 3D.

To understand the dynamics of CDA we applied our results to the Smoluchowski aggregation equation (generalized to include cell replication) and derived the mean rate of aggregation of two clusters (also known as the coalescence kernel). This rate is proportional to the production of chemoattractant by both clusters and to the chemotactic coefficient *χ*(*c*), and is inversely proportional to the diffusion coefficient *D*. The aggregation time, which is calculated by plugging the expression for the kernel into the Smoluchowski equation (see SI text) reads:
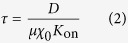
which is independent of initial density, as found in the experiments. This result follows directly from the counteracting effects of pairwise coalescence and chemotaxis. On the one hand coalescence is more probable whenever clusters are closer and thus denser. On the other hand the denser the clusters the slower their speed due to Weber-Fechner response (1). The balance of these two aspects makes the aggregation time independent of cell density.

### Numerical simulations

To gain further insights on CDA dynamics, we performed numerical simulations of the aggregation process. The numerical approach has a twofold motivation: on the one hand it allows to check several approximations that were made in the theoretical formulation of the model (see SI text), on the other hand, it allows exploration of different parameter sets, thereby giving a comprehensive view of how CDA might represent for cancer cells an efficient strategy to invade the surrounding space.

Results of our simulations are shown in Fig. 3C–E. In our theoretical modeling of CDA above, we have adopted a mean-field point of view, i.e. we have described the dynamics of a representative pair of clusters. While this approach is clearly advantageous as it allows to obtain predictions for many-body problems that would otherwise be unaccessible, its validity has to be tested. Numerical simulations of CDA show indeed that the theory developed above is an extremely good approximation of the dynamics of a population of clusters, as shown in Fig. 3F. Therefore we have checked whether the theoretical predictions obtained for the distribution of the velocity at early aggregation stages is correct, and we have found agreement between experiments, theory and numerical simulations.

As a particular case, we have investigated the situation where proliferation depends on the local concentration of secreted factor felt by each cluster. This corresponds, for example, to the case where cells secrete two factors, one responsible for cell migration and the other for cell proliferation – or when one single factor mediates both effects. We assume that the proliferation response of each cell to the mitogenic growth factor is increasing up to a reference concentration and then decreases, as suggested by our data in Fig. 1F,G. Under this assumption, the mean cell proliferation rate in an aggregation assay exhibits a bell-shaped behavior in time similar to the one observed in Fig. 3G. Note that, even though a concentration-dependent proliferation induces a feedback on migration through changes in the amount of secreted factor, the aggregation kinetics appears to be largely unchanged. (not shown).

### PC3 cells perform chemotaxis toward their own culture conditioned media

**Figure 4:**
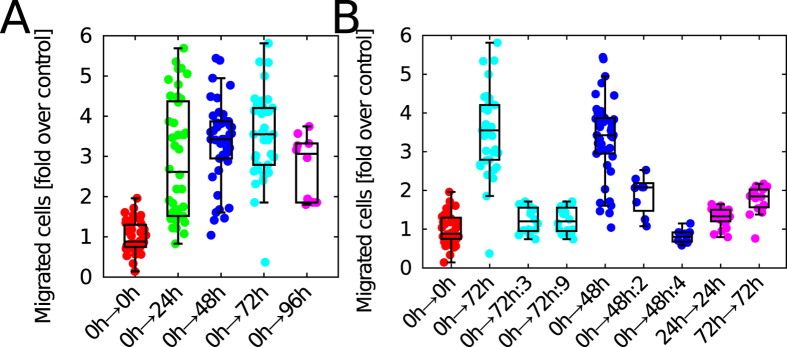
Chemotaxis of PC3 cells to autocrine secreted factors. (**A**) To assess the ability of PC3 conditioned media to induce chemotaxis of PC3 cells we collected the conditioned media in serum free media over different times. We then used the PC3 culture conditioned media in a classical chemotaxis assay. Migration of cells from serum free media (indicated as 0 h) towards serum free media was used as a control. Conditioned media collected at 24 h, 48 h, 72 h and 96 h is increasingly effective in inducing chemotaxis of PC3 cells, with the experimental point at 96 h showing a slight decreased effect. For the sake of comparison we performed migration of PC3 towards 0.5% FBS and obtained a median of 6.9 (fold over 0 h migrating towards 0 h). The number of migrated cells is normalized with the number of migrated cells in the case of serum free (0 h → 0 h, 50.0 ± 3.5 cells, median ± SEM). Statistical significance was assessed by means of a two-tailed t-test. Control vs any of the conditioned media yields a p-value smaller than 10^−10^. 96 h against 72 h yields a p-value smaller than 0.05. The box-plot indicates median, first and third quartiles and the whiskers extend over the respective quartiles for a length equivalent to 1.5 inter-quartiles length. Each point represents one field of view. (**B**) To verify whether the migration effect was genuine we performed a set of control experiments. We diluted the conditioned media collected at 72 hours 3 and 9 fold (72 h:3 and 72 h:9 respectively) and found that with this dilution cells migrate as in the control. The same experiment was repeated with the conditioned media collected at 48 h and diluted 2 and 4 fold (48 h:2 and 48 h:4 respectively). Furthermore to exclude purely chemokinetic or proliferative effects we repeated the same experiments by adding the same medium (i.e. 24 h or 72 h) in both the upper and lower transwell chambers. We obtained that indeed the conditioned media has a genuine effect as its presence in both the chambers did not induce migration as in the previous conditions. As a reference the same experiment performed with 0.5% FBS in both chambers gives a median of 2.26.

To verify whether our hypothesis on the mechanisms of CDA could be true, we collected conditioned media from PC3 cultures and assayed the cells ability to perform chemotaxis towards their own conditioned media. Indeed conditioned media collected in serum deprived cultures is able to attract cells (see Fig. 4A) and its effect is concentration dependent as shown in Fig. 4B, e.g. dilution of the media of a factor 3 stopped the chemotactic effect. Furthermore, to test whether the factor was accumulated over time, we tested different batches of conditioned media collected at 24, 48 72 and 96 hrs of culture and obtained an increasing number of migrated cells over the control. Results are shown in Fig. 4A,B. As further controls we assayed the ability of PC3 cells to perform chemotaxis towards conditioned media collected from cultures of other prostate lines finding that indeed, although to a lesser extent, DU145 and LnCaP conditioned media attract PC3 cells. On the other hand, LnCaP cells were not able to migrate effectively towards their own conditioned media (Fig. SI2A-B). Taken together these data indicate that PC3 secrete a chemotactic factor and support the CDA hypothesis as an explanation for the aggregation of PC3 cells.

## Discussion

In this paper we presented experimental evidence showing a novel phenotype of CCs. Our results indicate that when embedded in a BME gel basement membrane, CCs can grow as spheroids and aggregate forming larger and larger structures. We quantified the dynamics of the aggregation process and found that the aggregation time is independent of seeding density. Conversely, our measurements of cell velocity at the early stage of aggregation indicate that the average velocity depends on seeding density. Theoretical arguments show that the observed behavior is not consistent with aggregation due to the random motion of cells and point to a long-distance interaction between clusters. We formulated a theoretical model of chemotaxis-driven cell aggregation and found very good agreement between our experimental data and the theoretical predictions. We confirmed the plausibility of CDA in PC3 by proving their ability to migrate towards conditioned media in a chemotaxis assay.

One possible explanation for the evolutionary emergence of CDA is the assumption that the diffusible attractant is also a growth factor. Autocrine loops are ubiquitously found in cancer^3^,^35^ and the same holds for chemotaxis-related genes and phenotypes. Since it is not unusual to find growth factors to have impact on both migration and proliferation, an interesting issue is whether a cell might actually gain simultaneously the ability to migrate faster, in a directional way and to replicate faster than its neighboring cells. This hypothesis would also suggest that, since the concentration of chemoattractant is higher where cell clusters are bigger, CDA would also strongly increase cell proliferation in a positive feedback loop, thereby conferring an even larger selective advantage to cells that aggregate. A simple theoretical argument supporting this hypothesis is presented in the Supplementary Material.

In our study we have not considered the effect of mechanical deformation of the matrix nor that of matrix degradation and subsequent variation of mechanical properties. While these aspects are with no doubts of clear importance in cancer invasion in general, their effect on aggregation would be secondary as it would impact local cluster-cluster interactions, but would not hinder diffusion or drive aggregation per se. Several cell lines have been reported to undergo homotypic or heterotypic aggregation in liquid cultures *in vitro*. However within such culture conditions, cells enter in contact due to passive physical mechanisms and exploit cell-adhesion molecules to form multi-cellular clusters^26^,^36^,^37^,^38^,^39^,^40^. Therefore, while our assay shows the prowess that cells display in actively searching for other cells, aggregation in liquid overlay cultures reflects the ability of cells of staying together upon contact, and is therefore a completely different phenomenon.

PC3 cell line is long known for its ability to form spheroids and to invade BMEs (see^29^,^41^,^42^,^43^,^44^ and refs. therein) and has been recently reported to migrate collectively in a N-cadherin dependent fashion^45^. However it is not known whether the ability of growing as spheroids and that of migrating collectively can act synergistically to improve the cells ability to invade or grow. Potentially, CDA might confer selective advantage in two separate ways: i) the local concentration of secreted growth factors and proteases around a cell aggregate is high, thereby yielding potentially increased invasiveness and proliferation and ii) cells that do not possess the autocrine loop but do express the receptor for the factor secreted by other cells could perform a sort of hitchhiking thus resulting in a larger clonal heterogeneity. Both these aspects might contribute to the overall survival of cancer clones. Interestingly, prostate cancer (the origin of the cell line used in this study) is known to be often multifocal^46^,^47^,^48^, thereby making such aspects potentially relevant in this context.

An interesting issue is whether CDA might be relevant in the context of primary tumor site, in the spreading of cancer cells toward distant sites or even in the secondary site. While a role for tumor multicellular clusters has been hypothesized in the metastatization process^25^,^27^, its advantage in colonizing a primary or distant site is less obvious and remains to be proven.

Our work describes CDA as a novel mechanism of growth in cancer. This phenotype is curiously opposed to what happens in other contexts where cancer development is based on detachment of single cells from a multicellular structure and migration regardless of cell-cell or cell-matrix adhesion signals. A role for CDA *in vivo* still remains to be proven. Nevertheless our theoretical and experimental results point at a general relevance for this process, and unveil an additional mechanism by which cancer cells might divert otherwise physiological functions and abilities for their own purpose and benefit.

## Additional Information

**How to cite this article**: Puliafito, A. *et al.* Three-dimensional chemotaxis-driven aggregation of tumor cells. *Sci. Rep.* **5**, 15205; doi: 10.1038/srep15205 (2015).

## Supplementary Material

Supplementary Information

Supplementary Movie 1

Supplementary Movie 2

Supplementary Movie 3

Supplementary Movie 4

Supplementary Movie 5

## Figures and Tables

**Table 1:** Model parameters.

**Parameter**	**Definition**	**Value**
*n*	initial cell density	10 ÷ 100 mm^−3^
*a*	cell diameter	20 *μ*m
*α*	cell proliferation rate	0.02 h^−1^
*β*	production rate of chemoattractant per cell	100 ÷ 1000 molecules *s*^−1^
*χ*_0_*K*_on_	chemotactic response	0.5 *μ*m^2^ s^−1^
*μ*	chemoattractant degradation rate	0.1 h^−1^
*D*_*c*_	chemoattractant diffusivity	10 *μ*m^2^ s^−1^

The values of *n, a* and *α* were directly estimated from experimental images.

The value of the diffusion coefficient *D*_*c*_ was taken from refs 49, 50 and represents a typical value of diffusion coefficient of a growth factor. The value of *μ* was estimated by imposing an interaction length of roughly 600 *μm*, as measured in aggregation assays. The chemotactic response was estimated by imposing a typical velocity of 1 ÷ 10 *μm*/*h*, by using eq. (1) with a relative gradient 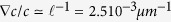. *β* was estimated by imposing that the average concentration at working cell/clusters concentrations to be 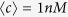, and by using eq. (29) of the SI text.
