# Supplementary material for: Three-dimensional chemotaxis-driven aggregation of tumor cells
Source: Sci Rep. 2015 Oct 16;5:15205. doi: 10.1038/srep15205 (PMC4607978; doi:10.1038/srep15205)
Supplement: Supplementary Information [file srep15205-s1.pdf]

Three-dimensional chemotaxis-driven aggregation of tumor cells
SUPPLEMENTARY INFORMATION

Alberto Puliafito[1], Alessandro De Simone[2], Giorgio Seano[1,3], Paolo Armando Gagliardi[1,4], Laura Di Blasio[1,4], Federica Chianale[1], Andrea Gamba[5,6,7], Luca Primo[1,4], and Antonio Celani[8]

[1] *Candiolo Cancer Institute FPO-IRCCS, Candiolo, Turin, Italy*
[2] *Swiss Institute for Experimental Cancer Research (ISREC),School of Life Sciences, Swiss Federal Institute of Technology (EPFL) Lausanne, Switzerland*
[3] *Edwin L. Steele Laboratory for Tumor Biology, Harvard Medical School, Massachusetts General Hospital, Boston, MA, USA*
[4] *Department of Oncology, University of Turin, Turin 10060, Italy*
[5] *Institute of Condensed Matter Physics and Complex Systems, Department of Applied Science and Technology, Polytechnic University of Turin, Corso Duca degli Abruzzi, 24, 10129 Torino, Italy*
[6] *Human Genetics Foundation (HuGeF), Via Nizza 52, Torino, Italy*
[7] *Istituto Nazionale di Fisica Nucleare (INFN), Torino, Via Giuria 1, 10125 Torino, Italy*
[8] *Quantitative Life Sciences Unit, The Abdus Salam Center for Theoretical Physics (ICTP), Strada Costiera 11, I-34151 Trieste, Italy*

Contents

1 Dynamics of the chemoattractant receptor **2**

2 Chemotactic model **5**
- 2.1 The chemoattractant concentration field 6
- 2.2 Distribution of the chemoattractant concentration 6
- 2.3 Distribution of the chemoattractant gradient 8
- 2.4 Large gradients are determined by nearest-neighbor interaction . 9
- 2.5 The contribution of farther clusters 9
- 2.6 Distribution of velocities 11

3 Cluster proliferation-aggregation model **11**
- 3.1 Chemotactic kernel 12
- 3.2 Chemotactic aggregation 12

4 Advantage of aggregating vs non-aggregating cells. 13

5 Cluster proliferation-aggregation computational model 14
5.1 Periodic boundary conditions 15

6 Supplementary Experimental Results 17

7 Supplementary Movies legends 18

Synopsis

Supplementary material presents more detailed information on the results discussed in the Main Text, following the plan described hereafter.

In Sec. 1, we derive the chemotactic response that we have considered, by studying the microscopic ligand-receptor dynamics in presence of a chemoattractant gradient. In *2*, we define the chemottractant dynamics and the cluster chemotactic response. We focus on the chemoattractant statistics and on the distribution of cluster velocities. We show that the chemoattractant concentration results from the contribution of all the clusters within the interaction distance and is uniform in space. Thus, the velocity probability distribution depends only on the statistics of the chemoattractant gradient. In particular, we show that the distribution of velocities has heavy tails due to the interaction with the nearest cluster.

In *3*, we propose an aggregation-proliferation analytic model based on macroscopic quantities such as the density of clusters of given number of cells. By using the chemotactic response previously described, we solve the analytic model for relevant observables such as cluster and cell number density. Sec. 4 contains a theoretical argument to illustrate why CDA might represent a selective advantage over non-aggregating cells. Furthermore, we compare the results of the analytic model with a computational simulation that is described in *5*.

In Sec. 6 we present further experimental evidence supporting the results of the main text and describe the corresponding Materials and Methods.

1 Dynamics of the chemoattractant receptor

The interaction between ligand and receptor is the first step in the signaling cascade that leads to cell response. The response to chemical stimuli is assumed to be proportional to the number of actively signaling receptors on the cell (see [1] and [2]).

In our model a gradient of chemoattractant, i.e. the ligand, modifies the distribution of bound receptors on cell surface. As a result of this asymmetry, the cell moves in the direction of the gradient.We approximate the cell with a sphere of radius a characterized by a surface density of receptors R_{s} which

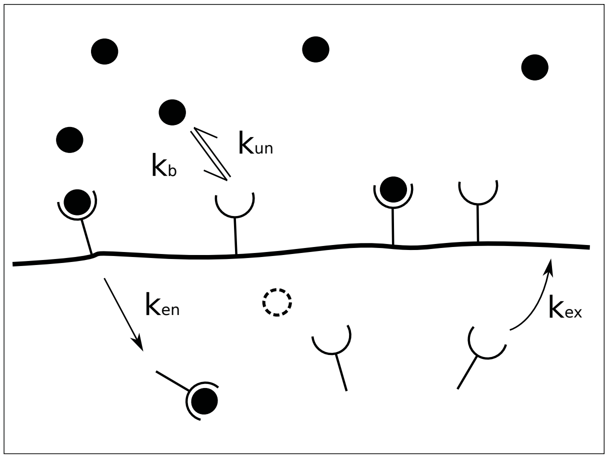

Figure 1: Representation of the receptor dynamics. $k_{\rm b}$ is the receptor-ligand binding rate, $k_{\rm un}$ is the unbinding rate. A bound receptor can be internalized by the cell with an endocytosis rate $k_{\rm en}$ and $k_{\rm ex}$ is the number of free receptors per unit time that exocytosis brings to the surface of the cell. Adapted from [1]

is the sum of a surface density $R_{\rm un}$ of unbound receptors and $R_{\rm b}$ of bound receptors. The surrounding cells produce a ligand that diffuses with a diffusivity D_c and its concentration profile rapidly reaches the equilibrium. We consider a gradient of ligand $g = |\nabla c|$. The concentration at large distances from the other clusters is:

$$c(r,\theta) = c_0 + gz = c_0 + gr\cos\theta \tag{1}$$

where we have used spherical coordinates with zenith direction z and inclination θ, and approximated the gradient by means of Taylor expansion. The system is invariant for rotations along the azimuth ϕ.
We suppose that the gradient g is small, so that ga, i.e. the variation of concentration within the cell length, is small in comparison with the concentration c_0. We consider a first-order dynamics where $k_{\rm b}$ is the receptor-ligand binding rate and $k_{\rm un}$ is the unbinding rate. A bound receptor can be internalized by the cell with an endocytosis rate $k_{\rm en}$. Inside the cell a complex system of vesicles and enzymes leads to unbinding of receptors, to their recycling or degradation, possibly with the synthesis of new receptors. We simplify the description assuming that exocytosis brings $k_{\rm ex}$ free receptors per unit time to the surface of the cell. The whole receptor dynamics is graphically sketched in Fig.S1. The mass-action kinetics is given by the system of differential equations

$$\frac{dR_{\rm b}}{dt} = k_{\rm b}c(r,\theta)R_{\rm un} - k_{\rm un}R_{\rm b} - k_{\rm en}R_{\rm b} + \frac{D_s}{a^2\sin\theta}\frac{\partial}{\partial\theta}\sin\theta\frac{\partial R_{\rm b}}{\partial\theta} \tag{2}$$

$$\frac{dR_{\rm s}}{dt} = -k_{\rm en}R_{\rm b} + \frac{k_{\rm ex}}{4\pi a^2} + \frac{D_s}{a^2\sin\theta}\frac{\partial}{\partial\theta}\sin\theta\frac{\partial R_{\rm s}}{\partial\theta} \tag{3}$$

where we have introduced a diffusivity D_s of the receptors on the surface of the cell. Since we suppose that the gradient g varies slowly with respect to the receptor dynamics timescales, we study the equilibrium solution. The solution of the diffusion equation with the boundary condition eq. (1) is

$$c(r,\theta) = c_0 - \frac{A_1}{r} + \left(r - \frac{A_2}{r^2}\right) g\cos\theta \tag{4}$$

where the coefficients A_1 and A_2 are set by the conservation of ligand at cell surface

$$D_c \left.\frac{\partial c}{\partial r}\right|_{r=a} = k_{\rm b} c(a,\theta) R_{\rm un} - k_{\rm un} R_{\rm b} \tag{5}$$

In order to simplify the equations, we rescale the variables with the total number of receptors in the cell $R_{\rm tot}$. Then, we consider the variables $r_{\rm b} = R_{\rm b}/R_{\rm tot}$ and $r_{\rm s} = R_{\rm s}/R_{\rm tot}$. We introduce the characteristic concentrations

$$K_1 = \frac{k_{\rm en}}{k_{\rm b}} \qquad K_2 = \frac{k_{\rm ex}}{4\pi D_c a} \tag{6}$$

$$K_3 = \frac{D_s}{a^2 k_{\rm b}} \qquad K_4 = \frac{k_{\rm ex}}{4\pi a^2 R_{\rm tot} k_{\rm b}} \tag{7}$$

$$K_d = \frac{k_{\rm un}}{k_{\rm b}} \tag{8}$$

Equations (2),(3),(5) at equilibrium become

$$c(a,\theta)(r_{\rm s} - r_{\rm un}) - K_d r_{\rm b} - K_1 r_{\rm b} + K_3 \frac{1}{\sin\theta}\frac{\partial}{\partial\theta}\sin\theta\frac{\partial r_{\rm b}}{\partial\theta} = 0 \tag{9}$$

$$K_1 r_{\rm b} + K_4 + K_3 \frac{1}{\sin\theta}\frac{\partial}{\partial\theta}\sin\theta\frac{\partial r_{\rm s}}{\partial\theta} = 0 \tag{10}$$

$$a\frac{K_4}{K_2}\left.\frac{\partial c}{\partial r}\right|_{r=a} - c(a,\theta)(r_{\rm s} - r_{\rm b}) + K_d r_{\rm b} = 0 \tag{11}$$

We solve this system of ordinary differential equations perturbatively. We study the solutions at the lowest order in $\frac{ga}{c_0} \ll 1$ that is

$$r_{\rm b} = r_{\rm b}^0 + g\cos\theta\, r_{\rm b}^1 \tag{12}$$

$$r_{\rm s} = r_{\rm s}^0 + g\cos\theta\, r_{\rm s}^1 \tag{13}$$

The solution in absence of gradient is isotropic and the system shows perfect adaptation, i.e. the equilibrium surface density of bound receptors is independent of the chemoattractant concentration:

$$r_{\rm b}^0 = \frac{K_4}{K_1} \tag{14}$$

and the total surface density of receptors is

$$r_{\rm s}^0 = \frac{(c_0 + K_1 - K_2 + K_d)}{c_0 - K_2} r_{\rm b}^0 \tag{15}$$

Since in general $r_\mathrm{b} < r$, one has that $K_2 < c_0$ in order to ensure a valid solution. Solving the equations for the first-order perturbation, one has

$$r_\mathrm{b}^1 = \frac{6aK_1K_3(K_1+K_d)(c_0K_1+K_2K_d)r_\mathrm{b}^0}{\Big(2c_0K_1+K_2(-K_1+K_d)\Big)} \times \\ \times \Bigg(c_0^2K_1(2K_3-K_1) - K_2\big(K_1^2K_2 - 2K_1K_2K_3 + 4K_3^2K_d\big) + \\ + 2c_0K_1\Big(K_1(K_2+K_3) + K_3(2K_2+2K_3-K_d)\Big)\Bigg)^{-1} \quad (16)$$

For $K_2 \ll c_0 \ll K_3$ and $\frac{K_d}{K_1}K_2 \ll c_0$ the response to the gradient is

$$r_\mathrm{b}^1 = \frac{3}{2}a\frac{(K_1+K_d)r_\mathrm{b}^0}{(K1+K_d-2K_3)}\frac{1}{c_0} \propto \frac{1}{c_0} \quad (17)$$

Finally, assuming that the chemotactic response is proportional to the perturbation of the density of bound receptors, one has

$$v \propto gr_\mathrm{b}^1 \propto \frac{\nabla c}{c_0} \quad (18)$$

2 Chemotactic model

We propose a model in which aggregation is driven by chemotaxis. We suppose that each cell in a cluster produces a diffusible chemoattractant that generates a concentration profile around the cluster itself. Cells measure the chemoattractant concentration through receptors on their surface and respond by moving toward the source, along the concentration gradient.

We suppose that each cell in a cluster responds in the same way to chemical stimuli, so that the cluster moves as a whole under the action of the chemoattractant gradient. In general the chemotactic response depends on ambient concentration. Here we assume the following form for the velocity

$$\mathbf{v} = \chi_0 \frac{\nabla c K_\mathrm{on} K_\mathrm{off}}{(c+K_\mathrm{on})(c+K_\mathrm{off})} \quad (19)$$

where χ_0 is a reference chemotactic responsivity and K_on, K_off are reference concentrations. This expression have been derived in *1*. For small values of concentration, $c \ll K_\mathrm{on}$, the dependence is $v \propto \nabla c$, while for $c \gg K_\mathrm{off}$ is $v \propto \frac{\nabla c}{c^2}$. The intensity of the response for intermediate values is

$$\mathbf{v} \propto \frac{\nabla c}{c} \quad (20)$$

In this case the response is proportional to the relative variation with respect to the background value.

2.1 The chemoattractant concentration field

We suppose that each cell produces independently the chemoattractant at a rate β, so that the production of each cluster is proportional to its number of cells. Moreover, since cell motility is relatively slow with respect to molecular diffusion, the concentration profile rapidly reaches equilibrium. The stationary diffusion equation then gives

$$D_c \nabla^2 c + \beta \sum_{i=1}^{N} m_i \delta(\mathbf{r} - \mathbf{r}_i) - \mu c = 0 \tag{21}$$

where c is the chemoattractant concentration, $\mathbf{r}_i$ are the positions of the sources, β is the cell chemoattractant production rate, D_c is the diffusion constant and μ is the chemoattractant degradation rate. We remark that in this context we use the variable m_i to indicate the number of cells in the i-th cluster. Since the clusters are approximately spheroidal and their size is negligible with respect to their distance, the chemoattractant production is considered as concentrated in their center of mass.

By solving equation (21) with the Green's function method, we obtain the concentration profile outside the clusters

$$c(\mathbf{r}) = G \sum_{i=1}^{N} \frac{m_i}{|\mathbf{r} - \mathbf{r}_i|} e^{-\frac{|\mathbf{r}-\mathbf{r}_i|}{\lambda}} \tag{22}$$

with

$$G = \frac{\beta}{4\pi D_C} \qquad \lambda = \sqrt{\frac{D_c}{\mu}} \tag{23}$$

where λ is the interaction length-scale of chemotaxis. For example, assuming a diffusivity of the chemoattractant $D_c \approx 10\,\mu\mathrm{m}^2/\mathrm{s}$ and a degradation time μ^{-1} of 10 hours, the interaction length is $\lambda \approx 600 \mu m$.

2.2 Distribution of the chemoattractant concentration

The spatial distribution of chemoattractant is determined by the position of clusters. We consider an uniform distribution of clusters in a spherical volume V of radius R

$$n(\mathbf{r}, m) = \tau(m) \frac{1}{\frac{4}{3}\pi R^3} \tag{24}$$

where $\tau(m)$ is the distribution of cluster size, i.e. number of cell. We define the random variable g of the concentration field generated by a single cluster in the center of the volume

$$g = G \frac{m}{r} e^{-\frac{r}{\lambda}} \tag{25}$$

Then, by summing up the contributions of all the uniformly distributed clusters:

$$c = \sum_{i=1}^{N} g_i \tag{26}$$

The mean value of the single contribution is

$$\langle g \rangle = 4\pi G \lambda^2 \frac{\langle m \rangle}{V} \left(1 - \frac{R}{\lambda} e^{-\frac{R}{\lambda}} - e^{-\frac{R}{\lambda}} \right) \tag{27}$$

where $\langle m \rangle$ is the average cluster size. The second moment of the variable g is

$$\langle g^2 \rangle = 2\pi G^2 \lambda \frac{\langle m^2 \rangle}{V} \left(1 - e^{-2\frac{R}{\lambda}} \right) \tag{28}$$

Therefore c is the sum of N independent identically distributed variables with finite mean value and variance and the central limit theorem can be applied. As a result c is distributed as a Gaussian with mean value $\langle c \rangle = N\langle w \rangle$ and variance $\sigma_c^2 = N(\langle w^2 \rangle - \langle w \rangle^2)$. Finally, one can take the infinite volume limit $R \to +\infty$, while keeping constant the density of clusters $n = \frac{N}{V}$, to obtain

$$\langle c \rangle = 4\pi G M \lambda^2 = \frac{M\beta}{\mu} \tag{29}$$

$$\sigma_c^2 = 2\pi G^2 M_2 \lambda \tag{30}$$

with $M = n\langle m \rangle$ density of cells and $M_2 = n\langle m^2 \rangle$. The coefficient of variation of the concentration is

$$\frac{\sigma_c}{\langle c \rangle} = \left(\frac{1}{8\pi} \frac{\langle m^2 \rangle}{\langle m \rangle^2} \frac{1}{n\lambda^3} \right)^{1/2} \tag{31}$$

Over the experimental ranges of densities of clusters, considering $\langle m^2 \rangle / \langle m \rangle^2 \lesssim 10$, the standard deviation is always much smaller than the mean value. The average concentration is therefore approximately constant in space for all clusters configurations and equal to eq. (29). It is worth noting that here we assumed each cluster to be filled with cells, while in many contexts cancer cell spheroids are found to be hollow either because of some residual glandular morphology reminiscent of the epithelial origin or because of necrosis. However, the assumption that clusters are hollow would simply rescale all terms $\langle m \rangle$ into $3\langle m^{2/3} \rangle$, without affecting the dependence of the chemoattractant concentration on the cluster density.

The velocity of a cluster depends on the chemoattractant concentration that it senses, including the contribution by the cluster itself. This induces an additional term that can be evaluated as

$$c_i^{\text{self}} = \frac{\beta m_i}{4\pi D_c} \frac{1}{a_i} \tag{32}$$

Clearly, as the density of cells increases the self-interaction becomes negligible and the total concentration of chemoattractant is simply proportional to the

density of clusters. We conclude that the concentration level is mostly determined by the effect of distant clusters, as they are the majority. In the next section we will see that the concentration gradient is instead largely governed by the interaction with neighboring clusters.

2.3 Distribution of the chemoattractant gradient

We will now consider the distribution of the gradient

$$\nabla c(\mathbf{r}_i) = -G \sum_{i=1}^{N} m_i \left(\frac{1}{\lambda} + \frac{1}{r_i} \right) e^{-\frac{r_i}{\lambda}} \frac{\mathbf{r}_i}{r_i^2} \tag{33}$$

From the isotropy of the distribution of clusters it follows that

$$\langle \nabla c \rangle = 0 \tag{34}$$

and we can consider, without loss of generality, the component $u = \nabla_x c$ of the gradient, that is the sum of the contributions w due to each cluster

$$w = \frac{Gm}{r} \left(\frac{1}{\lambda} + \frac{1}{r} \right) e^{-\frac{r}{\lambda}} \cos\theta \tag{35}$$

The random variable w has infinite variance because of the divergence in $r = 0$ with $w^2 \sim r^{-4}$ and the central limit theorem cannot be applied. In this case it is necessary to resort to the generalized central limit theorem [3].

The distribution of u, sum of a large number of independent w variables, tends to a *stable distribution*, determined by the tails of $p(w)$. A symmetric stable distribution $p(w)$ has the asymptotic behavior

$$p(w) \sim \frac{\alpha w_*^{\alpha} \sin(\frac{\pi\alpha}{2}) \Gamma(\alpha)}{\pi |w|^{\alpha+1}} \tag{36}$$

and it is defined by the index α and the scale factor w_*. The tail of $p(w)$ is determined by the behavior of $w \simeq \frac{Gm}{r^2}$ for $r \to 0$

$$p(w) = \frac{2\pi}{5} \frac{G^{3/2} \langle m^{3/2} \rangle}{V} w^{-5/2} \sim w^{-5/2} \tag{37}$$

The velocity u thus follows a stable distribution of index $\alpha = \frac{3}{2}$, known as the *Holtsmark distribution*. The width parameter u_* grows with the number of clusters according to

$$u_* = N^{2/3} w_* \tag{38}$$

Comparing (36) and (37), from (38) one obtains

$$u_*^{3/2} = \frac{8\pi\sqrt{2\pi}}{15} G^{3/2} \langle m^{3/2} \rangle n \tag{39}$$

where n is the density of clusters.

2.4 Large gradients are determined by nearest-neighbor interaction

In the previous section we have calculated the exact distribution of the gradient for an uniform distribution of clusters. We show here that the tails of the distribution $p(u)$ are determined by the interaction with the closest cluster. The distribution of the position of the nearest neighbor is [4]

$$p_{\rm nn}(r_1, \cos\theta_1) = 2\pi n r_1^2 e^{-\frac{4}{3}\pi r_1^3 n} \tag{40}$$

From this distribution we calculate that the average distance of the nearest neighbor is

$$r_1 = \frac{\Gamma(\frac{4}{3})}{(\frac{4}{3}\pi n)^{1/3}} \simeq 0.56\, n^{-1/3} \tag{41}$$

For a density of clusters of $100/\mathrm{mm}^3$, their average distance $r_1 \approx 100\,\mu\mathrm{m}$ is smaller than the interaction length $\lambda \approx 600 \mu m$ calculated above. Then we can approximate the velocity as:

$$u \simeq \frac{Gm\cos\theta_1}{r_1^2} \tag{42}$$

Under this approximation, we obtain for the distribution of gradient induced by the nearest neighbor

$$p_{\rm nn}(u) = \frac{1}{2Gm}\Big(\frac{4}{3}\pi n\Big)^{-2/3}\gamma\bigg(\frac{5}{3}, \frac{4}{3}\pi n\Big(\frac{Gm}{u}\Big)^{3/2}\bigg) \tag{43}$$

where $\gamma(s, x)$ is the lower incomplete Gamma

$$\gamma(s, x) = \int_0^x y^{s-1} e^{-y} dy \tag{44}$$

The asymptotic behavior of the distribution is

$$p_{\rm nn}(u) \sim \frac{2\pi}{5} n (Gm)^{3/2} u^{-5/2} \tag{45}$$

that coincides exactly with the asymptotic behavior of $p(u)$. This agreement means that large values of u are entirely due to the interaction with the nearest neighbors. As shown in the Main Text, this approximation ceases to be valid for intermediate and low gradients.

2.5 The contribution of farther clusters

Since the nearest neighbor approximation captures the large-velocity events only, it is necessary to take into account the other contributions in order to

improve the approximation. The gradient can be split in two terms, the first one due to the nearest neighbor, the second one to the other clusters

$$u = \frac{Gm}{r_1^2}\cos\theta_1 + \tilde{u} \tag{46}$$

where

$$\tilde{u} = \sum_{i=2}^{N} \frac{Gm\cos\theta_k}{r_k}\left(\frac{1}{\lambda}+\frac{1}{r_k}\right)e^{-\frac{r_k}{\lambda}} \tag{47}$$

The clusters contributing to $\tilde{u}$ are uniformly distributed outside a sphere of radius r_1, the position of the nearest neighbor. For the single contribution

$$\tilde{w_k} = \frac{Gm\cos\theta_k}{r_k}\left(\frac{1}{\lambda}+\frac{1}{r_k}\right)e^{-\frac{r_k}{\lambda}} \qquad k \geq 2 \tag{48}$$

the mean value $\langle\tilde{w_k}\rangle = 0$ and the variance $\langle\tilde{w_k}^2\rangle$ is finite. Thus, the central limit theorem can be applied. The total contribution of the outer clusters is distributed as a Gaussian with zero mean value and variance

$$\sigma^2_{\text{out}}(r_1) = \frac{4\pi}{3} nG^2\langle m^2\rangle\left(\frac{1}{r_1}+\frac{1}{2\lambda}\right)e^{-\frac{2r_1}{\lambda}} \tag{49}$$

The distribution of u is the convolution of the distributions of the two contributions

$$p(u) = \int_{-1}^{1} d\cos\theta \int_0^{+\infty} dr_1\, p_{\text{nn}}(r_1,\cos\theta_1)\times$$

$$\times \frac{1}{\sqrt{2\pi}\sigma_{\text{out}}}\exp\frac{\left(u-\frac{Gm\cos\theta_1}{r_1^2}\right)^2}{2\sigma^2_{\text{out}}} \tag{50}$$

At this stage no approximations have been done and equation (50) is exact. The generating function for u reads

$$\langle e^{iku}\rangle = \int_0^{+\infty} p_{\text{nn}}(r_1)\frac{\sin(\frac{kGm}{r_1^2})}{\frac{kGm}{r_1^2}}e^{-\frac{1}{2}k^2\sigma^2_{\text{out}}(r_1)} \tag{51}$$

where the integrations over $\tilde{u}$ and $\cos\theta_1$ have been performed. The fact that the two terms contribute to different regions of $p(u)$ suggests to approximate them as if they were independent.

$$\langle e^{iku}\rangle \simeq \left\langle\frac{\sin(\frac{kGm}{r_1^2})}{\frac{kGm}{r_1^2}}\right\rangle\langle e^{-\frac{1}{2}k^2\sigma^2_{\text{out}}(r_1)}\rangle \tag{52}$$

and with increasing degrees of approximation

$$\langle e^{iku}\rangle \simeq \left\langle\frac{\sin(\frac{kGm}{r_1^2})}{\frac{kGm}{r_1^2}}\right\rangle e^{-\frac{1}{2}k^2\langle\sigma^2_{\text{out}}(r_1)\rangle} \simeq \left\langle\frac{\sin(\frac{kGm}{r_1^2})}{\frac{kGm}{r_1^2}}\right\rangle e^{-\frac{1}{2}k^2\sigma^2_{\text{out}}(\langle r_1\rangle)} \tag{53}$$

The velocity u thus can be approximated as the the sum of the nearest neighbor interaction plus a Gaussian contribution. The variance of this second contribution is given by (49), where r_1 is replaced by the average position of the nearest neighbor. As shown in the Main Text this approximation gives a very good agreement over the whole range of gradients.

2.6 Distribution of velocities

On the basis of the previous calculations we can now consider the velocity of the clusters

$$\mathbf{v} = \chi_0 \frac{\nabla c \, K_{\rm on} K_{\rm off}}{(c + K_{\rm on})(c + K_{\rm off})} \tag{54}$$

As discussed above, the distribution of c is strongly peaked and the concentration can be taken uniform and not fluctuating. Since ∇c results the only fluctuating variable, we can consider

$$\mathbf{v} = \chi(\langle c \rangle) \nabla c \tag{55}$$

where $\chi(\langle c \rangle)$ is the chemotactic coefficient

$$\chi(\langle c \rangle) = \chi_0 \frac{K_{\rm on} K_{\rm off}}{(\langle c \rangle + K_{\rm on})(\langle c \rangle + K_{\rm off})} \tag{56}$$

Each component of $\mathbf{v}$ is distributed with a Holtsmark distribution of width

$$v_* = \chi(\langle c \rangle) u_* \tag{57}$$

where $\langle c \rangle$ and u_* are respectively given by (29) and (39).

We remark that since $\langle c \rangle \propto n$ and $u_* \propto n^{2/3}$, in the range $K_{\rm on} \ll \langle c \rangle \ll K_{\rm off}$ the characteristic velocity v_* is proportional to $n^{-1/3}$.

3 Cluster proliferation-aggregation model

A complete description of cluster aggregation should include the spatial and geometrical properties of the clusters, such as their position, shape, size and velocity. Here we focus initially on a mean field model whose relevant variables are the number n_i of clusters per unit volume of a given number of cells i.

The master equation describing the evolution of the system is a generalization of the Smoluchowski aggregation equation [5]

$$\frac{\partial n_i}{\partial t} = \frac{1}{2} \sum_{j<i} \mathcal{K}_{i,i-j} n_j n_{i-j} - \sum_j \mathcal{K}_{i,j} n_i n_j + \alpha n_{i-1}(i-1) - \alpha n_i i \tag{58}$$

The aggregation process is determined by the *kernel* $\mathcal{K}_{i,j}$, i.e. the average rate at which two clusters of i and j cells coalesce. We have considered a constant cell replication rate α.

The most important quantities for our purposes are the total density of clusters $N = \sum_i n_i$ and the total density of cells $M = \sum_i n_i i$ evolving as

$$\frac{dN}{dt} = -\sum_{i,j} n_i \mathcal{K}_{i,j} n_j \tag{59}$$

$$\frac{dM}{dt} = \alpha M \tag{60}$$

3.1 Chemotactic kernel

At the basis of the Smoluchowski equation there is the assumption that pairwise interaction is the most relevant one. We calculate here the kernel as the collision rate between two clusters.

Given two clusters of i and j cells, the interaction is defined by eq.(20) where we have taken $K_{\mathrm{on}} \ll \langle c \rangle \ll K_{\mathrm{off}}$. The chemoattractant concentration is uniform and proportional to the cell density, as calculated in *2.2*. The resulting velocity is

$$\mathbf{v}_i = \frac{\beta}{4\pi D_c} \chi(\langle c \rangle) j \frac{\hat{\mathbf{r}}}{r^2} \tag{61}$$

for short distances with respect to the interaction length λ. This assumption is justified by the fact that the interactions leading to collision take place between neighboring clusters.

We choose a reference system in the center of the cluster of size i and we impose at large distance a density $n_j(\infty)$ of clusters of size j. The equation for the the spatial distribution of the clusters of size j is

$$\frac{\partial n_j(\mathbf{r}, t)}{\partial t} + \nabla \cdot (n_j(\mathbf{r}, t)\mathbf{v}) = 0 \tag{62}$$

where $\mathbf{v} = \mathbf{v}_i + \mathbf{v}_j$ is the relative velocity of the clusters. Since we want to calculate the average collision rate, we study the stationary solution. By integrating the stationary equation on a spherical shell around the cluster i, one obtains that the flux of clusters across each spherical surface is conserved and given by

$$\phi = \frac{\beta}{D_c} \chi(\langle c \rangle)(i + j) n_j(\infty) \tag{63}$$

Therefore, we conclude that the aggregation rate of two clusters of number of cells i and j is

$$\mathcal{K}_{i,j} = \frac{\beta}{D_c} \chi(\langle c \rangle)(i + j) \tag{64}$$

3.2 Chemotactic aggregation

The chemotactic kernel (64) is now inserted into the master equation (58) to study the aggregative dynamics. In the range of concentration $K_{\mathrm{on}} \ll c \ll K_{\mathrm{off}}$

it takes the form

$$\mathcal{K}_{i,j} \simeq \frac{\beta}{D_c}\frac{\chi_0 K_{\rm on}}{\langle c\rangle}(i+j) = \frac{\mu}{D_c}\frac{\chi_0 K_{\rm on}}{M}(i+j) \tag{65}$$

where eq. (29) has been used. The equation for the density of clusters N (59) becomes

$$\frac{dN}{dt} = -\frac{\mu}{D_c}\chi_0 K_{\rm on} N \tag{66}$$

with solution:

$$N(t) = N_0 e^{-\frac{\mu}{D_c}\chi_0 K_{\rm on} t} \tag{67}$$

where N_0 is the initial density of clusters. Then, for this kernel, the aggregation time

$$\tau = \frac{D_c}{\mu\chi_0 K_{\rm on}} \tag{68}$$

is independent of the initial density of clusters. The equation for $M(t)$ has already been obtained in eq. (60). In the case of a constant replication rate α it has the solution

$$M(t) = M(0)e^{\alpha t} \tag{69}$$

It is important to stress that, thanks to the independence of the density of clusters from cell density, the aggregative dynamics does not depend on the replication rate.

4 Advantage of aggregating vs non-aggregating cells.

An interesting question is whether aggregation might represent an advantage over cells simply producing chemoattractant but without coalescing. This question can be answered by the following calculations.

The flux of molecules secreted by a spherical cluster of radius R is

$$\phi = 4\pi D_c R c(R) \tag{70}$$

where D_c is the diffusion coefficient. This expression follows from the solution of the diffusion equation $D_c\nabla^2 c = 0$ outside the sphere, with vanishing concentration at infinity.

Now assume that at the surface of the cluster there will be production of chemoattractant at a rate Π^+, and consumption at a rate Π^-. These two contributions read:

$$\Pi^+ = 4\pi R^2 \beta n_p \qquad \Pi^- = 4\pi R^2 k_{\rm b} n_a c(R) \tag{71}$$

where n_p and n_a are the surface densities of cells that are producing or absorbing the ligand, respectively. The other parameters are $k_{\rm b}$, the absorption rate, and β, the production rate of ligand, per cell.

At the stationary state, the flux equates the balance between production and consumption

$$\phi = \Pi^{+} - \Pi^{-} \tag{72}$$

As a result, the consumption rate of ligand at the surface per cell is given by

$$k_{\mathrm{b}} c(R) = \frac{\beta \frac{n_p}{n_a}}{1 + \frac{D_c}{R k_{\mathrm{b}} n_a}} \tag{73}$$

which grows with the cluster size R and saturates for $R \gg R^{*} = D_c/(k_{\mathrm{b}} n_a)$ to the value $\beta n_p/n_a$. Therefore, as long as the cluster's size is smaller or comparable to R^{*} the absorption of ligand *per cell* increases with the cluster's size.

The physical reason for such an enhancement resides in the properties of diffusion. Secreted molecules tend to bounce back to the surface with great probability: this results in the well known linear dependence of ϕ on the size R as opposed to the quadratic dependence for ballistic motion. Conversely, production and consumption do grow proportionally to the surface, i.e. as R^2, in this case. Increasing R mitigates the effect of ligand dispersion relative to production and thereby increases the efficiency of recapture. Indeed, as $R \gg R_{*}$ the ratio of consumption to production approaches unity.

This argument can be generalized to the case when production and consumption takes place also inside the volume. The calculations are more cumbersome and will not be reproduced here, but, as the intuition suggest, the argument above holds as well and the result is qualitatively similar.

Finally, it is only left to estimate R^{*} in order to check that is sufficiently large with respect to the cell size. For this purpose we use the following values:

$$D_c \sim 10\ \mu m^2/s \tag{74}$$

$$k_{\mathrm{b}} \sim 2 \cdot 10^6\ M^{-1} s^{-1} \tag{75}$$

$$1/n_a \sim 600\ \mu m^2 \tag{76}$$

where for n_a^{-1} we have used an estimation of about half the surface of a cell with a $10\mu m$ diameter, while D_c has the same estimation as in the paper and for k_{b} we refer to the values cited in ref. [1]. With these values, we obtain $R^{*} \sim 10^6$ which is four orders of magnitudes larger than the radius of a single cell.

5 Cluster proliferation-aggregation computational model

We propose a computational model in which clusters are treated as spheres of volume proportional to the number of cells. Initially clusters are uniformly distributed in a cubic box of side L. Clusters move as rigid bodies with a velocity

$$\mathbf{v} = \chi_0 K_{\mathrm{on}} \frac{\nabla c}{c} \tag{77}$$

where the concentration is measured in the center of mass of the clusters and it comprises the self-interaction term. At any time, the concentration measured by a cluster is

$$c_i = \frac{\beta}{4\pi D_c} \sum_{j \neq i} \frac{m_j}{|\mathbf{r}_j - \mathbf{r}_i|} e^{-\frac{|\mathbf{r}_j - \mathbf{r}_i|}{\lambda}} + \frac{\beta m_i}{4\pi D_c} \frac{1}{a_i} \tag{78}$$

as given by eq. (22). The $\mathbf{r}_j$ are the positions of the clusters and a self-interaction term is given by eq. (32).

Coalescence occurs when two clusters overlap. In this case the two clusters coagulate in a single one, located in the center of mass of the pair. We have used a constant replication rate. The parameters of the models are defined in Table 1 in the main text.

5.1 Periodic boundary conditions

Chemotactic aggregation is a typical many-body problem with long-range interactions. The computational cost for a single time-step scales as $O(N^2)$ where $N = \lambda^3 \sim 10^4$. These figures are clearly prohibitive, so that we opted for the alternative strategy of partitioning the interaction volume λ^3 in smaller boxes of volume L^3 and use periodic boundary conditions.

The concentration field produced by the clusters is the solution of eq. (21). The periodic Green function is a sum over all the lattice sites $\mathbf{r}_s$

$$g(\mathbf{r}, \mathbf{r}') = g(\mathbf{R}) = G \sum_s \frac{e^{-\frac{|\mathbf{R} - \mathbf{r}_s|}{\lambda}}}{|\mathbf{R} - \mathbf{r}_s|} \tag{79}$$

where $\mathbf{R} = \mathbf{r} - \mathbf{r}'$. The concentration is obtained integrating over the source distribution $\rho(\mathbf{r}')$ and in the case of point sources it is

$$c(\mathbf{r}) = \sum_i g(\mathbf{r} - \mathbf{r}_i) \tag{80}$$

with $\mathbf{r}_i$ position of the sources.

As the series in eq. (79) is slowly convergent, we resorted to the Ewald method, originally employed in solid state physics [6]. This method relies on the transformations of the series in eq. (79) in an alternative form of extremely fast convergence, by dividing appropriately the summation in coordinate and reciprocal spaces:

$$g(\mathbf{R}) = g_1(\mathbf{R}) + g_2(\mathbf{R}) \tag{81}$$

where

$$g_1(\mathbf{R}) = \frac{4\pi G}{L^3} \sum_n \frac{e^{i\mathbf{k}_n \cdot \mathbf{R} - \frac{1}{\eta}(|\mathbf{k}_n|^2 + \lambda^{-2})}}{|\mathbf{k}_n|^2 + \lambda^{-2}} \tag{82}$$

is a summation over reciprocal lattice vectors $\mathbf{k}_n$ and

$$g_2(\mathbf{R}) = \frac{2G}{\sqrt{\pi}} \int_{\frac{1}{2}\sqrt{\eta}}^{\infty} d\xi \sum_s \exp\left(-(\mathbf{r}_s - \mathbf{R})^2 \xi^2 - \frac{1}{4\xi^2 \lambda^2} \right) \tag{83}$$

is a summation in real space. The parameter η defines the way the sum is divided in the two terms and it has to be optimized to obtain the fastest convergent series.

The calculation of the integral of equation (83) leads to

$$\begin{aligned} g_2(\mathbf{R}) = \sum_s \frac{G}{2|\mathbf{R} - \mathbf{r}_s|} \Big(& e^{|\mathbf{R}-\mathbf{r}_s|/\lambda} \operatorname{erfc}\Big(|\mathbf{R} - \mathbf{r}_s| \frac{\sqrt{\eta}}{2} + \frac{1}{\sqrt{\eta}\lambda} \Big) + \\ & + e^{-|\mathbf{R}-\mathbf{r}_s|/\lambda} \operatorname{erfc}\Big(|\mathbf{R} - \mathbf{r}_s| \frac{\sqrt{\eta}}{2} - \frac{1}{\sqrt{\eta}\lambda} \Big) \Big) \end{aligned} \tag{84}$$

6 Supplementary Experimental Results

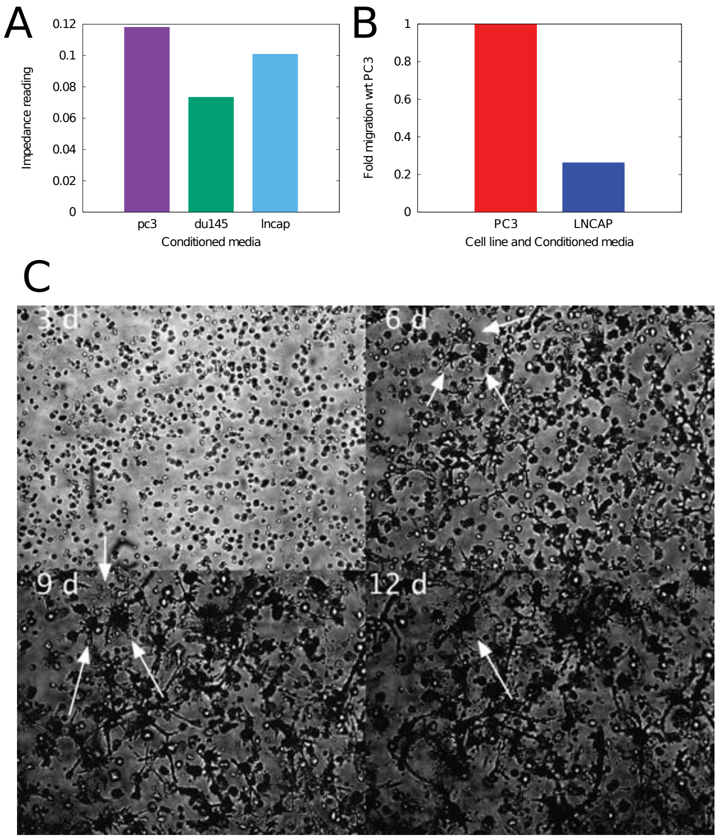

Figure 2: **A)** Migration of PC3 cells towards conditioned media collected from cultures of the three prostate cell lines PC3, DU145 and LNCAP. Conditioned media was collected at 24 hours. The migration assay is described in detail in the Supplementary experimental procedures of this document. Normalization is performed with respect to the signal with Serum Free media added both on top and bottom wells. **B)** LNCAP were tested in a migration assay towards their own conditioned media (collected at 24 hours). Each datapoint is normalized with respect to serum free media (as in panel A) and then to PC3 migration. **C)** Snapshots of MDA-MB-231 aggregating. White arrows highlight a group of clusters.

Supplementary experimental procedures

Migration assays presented in the supplementary material have been performed by means of the xCELLigence RTCA DP instrument (ACEA, San Diego, CA,

USA, details can be found in ref. [7] and refs therein) which was placed in a humidified incubator at 37°C and 5% CO2. Briefly, this instrument exploits a well-established technique based on the measurement of the frequency dependent electrical impedance of cell-covered electrodes subject to a small alternate electric current. Cells adhering on the electrodes vary the impedance in a frequency dependent manner. Migration is measured with a porous membrane analogous to Transwells which bottom is covered with electrodes measuring the presence of cells that migrated through the membrane (CIM-plate, ACEA, San Diego, CA, USA). 40000 cells were seeded for this experiment. Bars in the plot represent final points at 3h after seeding.

7 Supplementary Movies legends

SI Movie 1

PC3 cells were grown overnight in U-bottom multiwell plates and the culture media was supplemented with 1% methylcellulose, washed twice with PBS, resuspended in Matrigel and used in aggregation assay. The result of this procedure is to generate a broad distribution of cluster size.

SI Movie 2

Aggregation assay of PC3. Cells were detached from standard culture plates and resuspended as a single cell suspension into Matrigel at the desired concentration and put under the microscope. The result of this procedure is to generate a narrow size distribution, i.e. mostly single cells.

SI Movie 3

DU145 cells were processed as in SIMovie 2 and observed under the microscope.

SI Movie 4

LNCAP cells were processed as in SI Movie 2 and observed under the microscope.

SI Movie 5

MDA-MB-231 cells were processed as in SI Movie 2 and observed under the microscope.

References

[1] Lauffenburger D & Linderman J. ((1993); 2nd printing (1996)) *Receptors: Models for Binding, Trafficking, and Signalling*. (Oxford University Press).

[2] Wiegel F (1983) Diffusion and the physics of chemoreception *Physics Reports* **95**: 283–319.

[3] Kolmogorov A. N & Gnedenko B. V. (1968) *Limit Distributions for Sums of Independent Random Variables*. (Addison Wesley).

[4] Hertz P (1909) Uber den geigenseitigen durchschnitt- lichen abstand von punkten, die mit bekannter mittlerer dichte im raume angeordnet sind. *Mathematische Annalen* **67**: 387–398.

[5] Wattis J. A (2006) An introduction to mathematical models of coagulation-fragmentation processes: A discrete deterministic mean-field approach *Physica D: Nonlinear Phenomena* **222**: 1–20.

[6] Ham F. S & Segall B (1961) Energy Bands in Periodic Lattices–Green's Function Method *Phys. Rev.* **124**: 1786–1796.

[7] Gagliardi P. A, Puliafito A, di Blasio L, Chianale F, Somale D, Seano G, Bussolino F & Primo L (2015) Real-time monitoring of cell protrusion dynamics by impedance responses *Scientific reports* **5**.
